# Exploring professionalization among Brazilian oral health technicians

**DOI:** 10.1186/1478-4491-10-5

**Published:** 2012-07-12

**Authors:** Carla Aparecida Sanglard-Oliveira, Marcos Azeredo Furquim Werneck, Simone Dutra Lucas, Mauro Henrique Nogueira Guimarães Abreu

**Affiliations:** 1 Av. Antônio Carlos, Belo Horizonte, Minas Gerais, 6627 CEP 31270.901, Brazil

**Keywords:** Dental auxiliaries, Dental hygienists, Dental assistants, Professionalization

## Abstract

Professional dental auxiliaries emerged in the early 20th century in the United States of America and quickly spread to Europe and other regions of the world. In Brazil, however, oral health technicians (OHTs), who occupy a similar role as dental hygienists, had a long journey before the occupation achieved legal recognition: Brazilian Law 11.889, which regulates this occupation in the country, was only enacted in 2008. The aim of this paper is to review the literature on the professionalization of OHTs, highlighting the triggering, limiting and conflicting aspects that exerted an influence on the historical progress of these professionals in Brazil. We have tested Abbott’s and Larson’s theory on professionalization, against the history of OHTs. A number of different dental corporative interests exerted an influence over professionalization, especially in discussions regarding the permissible activities of these professionals in the oral cavity of patients. With primary health care advances in Brazil, the importance of these professionals has once again come to the forefront. This seems to be a key point in the consolidation of OHTs in the area of human resources for health in Brazil.

## Background

An oral health technician (OHT) is a dental auxiliary in Brazil. As with other auxiliary categories, they emerged in order to streamline dental work, increase productivity, increase quality and contribute toward scientific-technological development and changes in healthcare practices. The incorporation of these workers into health care services has allowed for an increase in patient coverage as well as changes in human resources in healthcare services [1].

OHTs are fundamental to the promotion of oral health as well as the prevention of and treatment of oral health diseases in both the public and private sectors on both the individual and population levels [2,3].

In Brazil, OHTs are part of the oral health team present in the “Family Health Team”, which was established with the consolidation of the public healthcare system [4]. These teams conduct their work based on the following principles: universality, equity and integrality [5] established by the Brazilian public healthcare system. The teams underlie the organizational strategy for healthcare services in Brazil [6]. There is a relatively international consensus on the advantage of public healthcare systems based on primary health care [7].

Despite the importance of OHTs, it was only in 2008 that a Brazilian federal Law 11.889 was enacted [8]. This Law has historical importance for OHTs.

The professional identity of OHTs (previously referred to as “dental hygiene technicians” - DHTs) remained compromised throughout much of the long history of the consolidation of this occupation in Brazil. As long as the activities of a particular worker are not regulated by legal devices, an open profile is associated with that type of worker, i.e. it is exempt from the principles that might establish an ideal professional position to be filled. In this case, any person could occupy the functions. According to the Brazilian constitutional principle regarding the free exercise of labour, trade or profession [9], a person need only meet the professional qualifications established by Law. If the Law does not establish these prerequisites, there are no legal grounds for the protection of those that acquire skills through courses and/or technical experience.

The aim of this paper is to review the literature on the professionalization of OHTs, highlighting the triggering, limiting and conflicting aspects that have exerted an influence over the historical progress of these professionals in Brazil. The research was performed using scientific papers indexed in the PubMed, Medline and Scielo databases as well as masters’ dissertations, doctoral theses and bibliographic material on the topic, such as book chapters and official legislation. The terms that were used in order to perform the literature review were the following: “oral health technician”, “dental assistants”, “dental auxiliaries”, “dental nurse” and “dental hygienists”. This study includes references spanning from 1949 to 2009, with the selection of material referring only to oral health technicians. The main objective of this article is to analyse the extent to which OHTs are professionalized in Brazil.

## Review and discussion

### Considerations on the process of professionalization

The process of professionalization of a particular occupational group is an issue explored especially in the sociology of professions. This field considers the social, economic and political relations that influence aspects such as the behaviour of the job market, the balance between the supply and demand of workers and the flexibility of employment ties [10].

A profession is defined as the mastery of a group over an organizational standard of labour. Moreover, there should be power and autonomy in the determination of who is qualified to perform a set of tasks in that a person should be able to control the criteria in order to control their own work. However, this power and autonomy only exist when legitimated by society and regulated by Law [11]. Thus, a profession is a self-regulated occupation that exercises a specialised activity founded on training and/or a specific education with a strong orientation toward the ideal of serving the community guided by ethical-professional principles. Therefore, the notion of a profession is intrinsically linked to the idea of a human activity that, through specialised knowledge, acts toward a particular “social purpose”. Self-regulation and autonomy prevail in this view and are two elements that allow a profession to have the “autonomy” to recreate social realities [12,13].

With regard to OHTs, these powers and issues regarding autonomy are even more complex due to the fact that their activities are supervised by another professional, the dentist. This type of inter-professional relationship is considered one of professional dependence on the part of those considered “outsiders” in relation to the authority of the established profession, which tends to maintain the ‘imprisonment’ of the former [14].

The central phenomenon defining the life of a professional is the link between the profession and their designated work range, referred to as “jurisdiction”. The exclusive rights for controlling a jurisdiction are gained through competition in distinct settings, or, “arenas”. Abbott [15] describes three main arenas in which professions should claim mastery over jurisdiction: (i) the arena of the legal system, which generally confers formal control over professional work; (ii) the arena of public opinion, in which professions construct images that influence and pressure the legal system in their favour; (iii) and the arena of the work setting, in which control of the field is actually performed and in which distortions may occur in regard to the official limits or jurisdiction imposed legally and publicly. In other words, what has been defined by Law may be adjusted in the workplace due to other cultural and social determinations. Thus, an important problem for any profession is the reconciliation of its public position with its position in the workplace. If the profession has not swayed public opinion enough, it will find it difficult to exercise mastery over its jurisdiction in the workplace, even when it is legally ensured the exclusive rights over this jurisdiction [15].

In analyzing dentistry’s jurisdiction in the context of OHTs, one may turn particularly to the OHT professionalization model drafted by Abbott [15] theory, which is based on the existence of a permanent dispute among professional jurisdictions with the aim of monopolizing a particular field of labor. The internal disputes within the field of dentistry occur between different occupational groups, especially because the jurisdiction is generally shared with subordinate occupations. These reason make it difficult to compare the theoretical framework of sociology of professions [11,12,15] with intermediate-level occupations. This could be because the monopoly concept is central to the professions and professionalization, and, in general, technical occupations are subordinated to professions that already hold professional monopoly. The regulatory demands of subordinate professionals do not necessitate technical autonomy and jurisdictional monopoly but rather the recognition and sharing of professional acts. As there is no specific theory to deal with these issues, it is important to collect and describe attributes from the OHT occupation as a starting point.

### The emergence of auxiliary human resources in dentistry worldwide and in Brazil

In the case of dentistry, there are at least two historical reasons for the development and expansion of auxiliary personnel in health services: the first is the “liberation” of ever more specialized professionals from simpler (but not less important) functions of dental work; and the second is related to the need to expand dental services at a lower cost due to increasing oral health problems. In recent years, the increase in the use of dental auxiliaries has been related to the growing use and diversification of preventive and educational measures. This change in paradigm, in which the aim is to establish and consolidate a new dental practice, the Oral Health Team, requires a professional with acquired skills to offer services in an active and dynamic fashion within a team. The efficiency of such a team (dentists and auxiliary personnel) depends on the skills of each of its members regarding the application of their personal qualifications, subordination of their personal interests and activity for the output of the team [16-18].

The emergence of dental auxiliaries may be as old as the practice of dentistry itself, but the formalization of auxiliary personnel occurred in the international context of the expansion of capitalism, the technical division of labour and specialization with the aim of the utilitarian optimization of increasing production along with a desire to lessen the work burden [1,19,20].

Alfred Fones demonstrated the benefits of the work of dental auxiliary staff with his own patients. Knowing that he would not be able to conduct all the necessary procedures, he trained his assistant to perform such tasks. Based on this experience, the first training program for young women, whom he called “dental hygienists”, was established at the Ohio College of Dental Surgery in 1910 [21,22].

The profile of dental auxiliaries in the world is extensive, but the use of such professionals was not always accepted with enthusiasm. There has often been controversy regarding their skills, the functions that should be conducted by these professionals and their required education [23-25].

In the United States of America, dental auxiliaries began to work with dentists in the 20^th^ century [21]. In 1907, a Law in the state of Connecticut formalized the occupation of the dental hygienist as an assistant to the dentist. This Law established the first dental auxiliary in the field of dentistry with juridical support, allowing specially trained individuals to examine, clean and polish teeth under the supervision of a dentist as well as to give instructions regarding oral hygiene [26].

The American experience led New Zealand to employ oral health professionals in the 1920s. Thus, the dental nurse emerged (currently referred to as a dental therapist) and, as in the United States of America, worked mainly with paediatric patients. Following a two-year training course, dental nurses worked in government programs directed at schoolchildren. However, their work had a broader scope than that of hygienists, as dental nurses also preformed restorations of teeth with carious lesions and administered local anaesthesia. While the American hygienists were essentially caregivers, the dental nurses could be considered operators under the indirect supervision of the dentist [27]. However, these titles are not equivalent around the world. For example, currently, in the United Kingdom, a dental nurse is the dental surgery assistant [28]. Dental hygienists and dental therapists are the only two clinical operators, apart from dentists, currently recognised legally in the UK. They are not permitted to conduct dental examinations or diagnoses [29].

With the support of the World Health Organization, the New Zealand model spread to different countries in Asia and Africa and even to South America. In 1927, a number of men in the Armed Forces of Paraguay were trained to prepare cavities, restore and extract teeth, administer local anaesthesia and perform other functions through a worker qualification program. These professionals were originally trained to treat military personnel under the supervision of a dentist. In the 1960s and 1970s, Colombia, Mexico and Venezuela also initiated this program but with a different nomenclature and different attributions. A number of countries in southeastern Asia also developed this human resource in the 1950s to deal with the oral health problems of their populations. In the following decades, there was a rapid expansion of the use of dental auxiliary personnel throughout the Americas, with different characteristics and use proposals, varying with the different countries and regions and with continued resistance on the part of the dental corporation [1,30].

There was also a type of auxiliary whose basic function was to hand the dentist instruments during dental care [31]. This professional, typically referred to as a dental assistant, emerged in the United Sates in the 1940s with the aim of augmenting care coverage. In Europe, the dental assistant also had a chaperone role [29].

In addition, in the United States of America, studies conducted in Woonsocket, Rhode Island in 1945 and Richmond, Indiana in 1946 addressed the activities of auxiliaries in oral health in the Incremental System, a series of programs for schoolchildren. These studies affirmed that the use of two auxiliaries, one alongside the dentist (“chair-side assistant”) and another mobile (“roving assistant”), who were in charge of ensuring the flow of patients, the sterilization work and other routine operations, would be the most efficient division of labour [32,33].

The experience in the state of Massachusetts (United States of America) in the 1950s reported by Dunning [23] is considered the benchmark for evaluating the function of technical auxiliaries. Dunning implanted a dental hygienist training program at the Forsyth Dental Infirmary for Children in the city of Boston. The various functions of these hygienists included inserting restorative material in cavities prepared by the dentist. Dunning claimed that if Richmond and Woonsocket enhanced productivity with the use of two auxiliaries (chair-side and roving), the introduction of a technical operator to restore previously prepared cavities would further enhance productivity by 50% or more.

Although the experience was restricted to the inner setting of an institution, the American Dental Association acted strongly to influence the prohibition of the experience, which occurred a few years later, despite the initial government incentives for studies on increasing productivity and the use of auxiliaries with a greater delegation of functions. Since the beginning of 20th century, dentists have made an effort to control their jurisdiction over dentistry [15].

Dunning [34] discusses the reasons for the resistance on the part of American dentists in accepting delegations for auxiliaries similar to those of the dental nurses, even when the latter had previously demonstrated technical success and gained the approval of New Zealand dentists. Dunning [34] states that a large number of American dentists were convinced that the short training period was limited and would provide inferior dental care, despite evidence to the contrary described in studies. The dentists also feared that the admission of auxiliaries with a short training period would constitute a threat to the professional autonomy or integrity of dentists (who would be responsible for the mistakes and poor practices of the auxiliaries). Dunning argues that, in order to circumvent this type of thinking, dentists needed only to learn to be captains of the team and excellent supervisors [34]. This was another example of dentists acting to maintain their jurisdictional monopoly over dentistry [15].

The American dental hygienist was, therefore, the first matrix for the organization of basic auxiliary models with direct action in the oral cavity: the hygienist, in the stricter sense, and the dental nurse or a blend of the two, known as “technicians”. The former is trained for oral health education and preventive measures (a “caregiver”), and the latter focuses mainly on clinical care but also works with education and prevention (an “operator”). It was the emergence of these dental auxiliaries that led to the germination of the OHT in Brazil.

The public healthcare situation in Brazil was precarious in the 1940s. More than 80% of the population lived in towns with less than 10 000 inhabitants, with little or no access to health and sanitation services. The majority of the population used untreated water. The lack of facilities for the adequate treatment and handling of sewage constituted a permanent risk factor for disease. In the large cities, where conditions were somewhat better, adequate services were only available to those who could afford them. Disease and death led to loss of human life, and there was a shortage of every type of healthcare professional [35].

In this national context, the Health and Sanitation Cooperation Program was established between Brazil and the United States of America in 1942. During World War II, the Brazilian Special Public Health Service (Serviço Especial de Saúde Pública – SESP) was created. The main goals of the SESP were to bring health services to the Amazon due to the scarcity of the strategic raw materials, rubber and iron ore, which were essential to the American war effort. Between 1950 and 1959, there was a greater territorial expansion of SESP to nearly the whole of Brazil [36].

The SESP public healthcare project was built on the training of healthcare personnel, education, the establishment of an integrated horizontal network of sanitary health units and the expansion of this network to state health departments. At first, it was intended to be a provisional agency responsible for specific sanitation policies. However, it lasted for 48 years. By 1960, SESP worked with autonomy from the Ministry of Health, a source of huge controversy. In the same year, the last contract with the United States of America expired, and the SESP was transformed into the Public Health Special Service foundation (Fundação Serviço Especial de Saúde Pública) [36,37].

Since the beginning of its activities, SESP was occupied with health education, concentrating its efforts on spreading information on the benefits of sanitation via posters, flyers and ad campaigns. Dental hygiene was part of the health education actions promoted by the institution [38].

In the 1950s, SESP promoted a profound change in dental care in the country and, to some extent, helped place oral health services on the agenda of public policies. The Incremental System was the main theoretical tool employed by sanitary dentistry for the diagnosis and treatment of oral health problems in the community. This method focused on the dental care of schoolchildren, addressing their accumulated needs and subsequently keeping those needs under control. The Incremental System followed priority criteria regarding age and oral health problems to indicate horizontal actions through a preventive program to control the incidence of oral diseases (especially caries) and vertical actions through curative programs to solve prevailing problems. At the same time, an educational program provided support to these actions [39,40]. Moreover, one of the presuppositions of the Incremental System was the training and use of auxiliary human resources.

The first Brazilian experience with dental auxiliary personnel occurred in the 1950s, when the SESP proposed that dental hygiene auxiliaries (DHAs) act in the control of caries through the topical application of fluoride and oral health education. This professional category was a legacy of the American auxiliary with a smaller number of delegated functions and was conceived as a type of assistant and hygienist hybrid, that did not perform the operating activities of dental nurses.

SESP was inspired by the incremental program of New Zealand, but did not follow the New Zealand pattern with regard to its auxiliaries. The agency preferred to train and use DHAs with far more limited functions due to caution and fear of the reaction of dentists. The topic was seen as taboo and the DHAs were only allowed in order not to give credence to accusations that SESP trained practical dentists [41,42]. At the time, Brazilian dentistry had established its arena [15], hindering the work of technical auxiliary personnel.

The use of DHAs was expected to enable the treatment and education of a greater number of patients and, along with allowing a proportional increase in the productivity of dentists within a given time unit, with substantial economy to the service. For the first time in Brazil, public services used the so-called “four-handed” dental technique. Although employed in a rudimentary fashion, the practice represented an advance at the time, as very few services used this method. In public dental services, the dentist generally worked alone, without adequate auxiliary labour support [43].

With its high point during the darkest days of the Brazilian military dictatorship (1968–1978), the Incremental System entered into decline in the 1980s. This system became ineffective as it was transformed into a routine. It became a pattern that was reproduced non-critically, and in the context of managerial precariousness, and the system was afflicted by a lack of resources and an absence of an epidemiological focus in the programs [25].

### OHTs of Brazil in the 1970s through to the present day

In the late 1970s, new alternatives to the Incremental System emerged, such as simplified dentistry, which followed the community (or simplified) medicine movement, with the aim of increasing productivity and broadening coverage at low costs through the simplification of techniques, equipment and human resources. The intention was to incorporate preventive and educational measures into curative measures in public health services, in an attempt to improve upon the Flexnerian model. This proposal offered novelties in the incorporation of alternative practices and teamwork, such as the incorporation of professionals, known at the time as DHTs, mainly in public services. At this point, there was an intensification of training of such personnel in Brazil that was encouraged by the Pan-America Health Organization [44,45].

The pressure from the population to obtain access to dental services made the different levels of the Brazilian government broaden the promotion of public dental services in the 1970s [44]. In 1971, law no. 5.692 led to the promulgation of elementary and high school teaching reform, which instituted new, universal and compulsorily professional high school teaching [46]. “Brazil needs technicians” was the motto of the educational policy of the dictatorship. The aim was for all high school students to obtain skills as technicians or technical auxiliaries. Thus, the role of containing the demand of candidates for higher education was given over to universal, compulsory professionalization in high school that would serve to send students directly to a job market supposedly lacking in skilled professionals, where they would be employed [47].

The origin of the DHT profession (formerly the OHT) in Brazil was in the bureaucratic-authoritarian hands of the military dictatorship. Ordinance no. 460/75 was drafted and defined by the Ministry of Education and Culture and the former Federal Board of Education. The ordinance instituted the definitions for the education of DHTs. These institutions described the attributes and essential requirements of DHTs for the exercise of their function as well as the training courses curricula [48]. The ordinance was important due to the “invention” of the profession of DHT. In this context, the DHT occupation was defined in a technocratic sense influenced by both the American and New Zealander experiences. At this time, although there was no imposition of a federal Law that recognised DHTs, the ordinance provided the basis of legal regulation of the DHT profession in Brazil.

In the 1980s, the Federal Council of Dentistry (FCD) defined the qualification guidelines for the DHT practices and the enrolment of these technicians in Regional Boards of Dentistry [49].

The content of this document offered different considerations for the justification of its issuance, arguing that after Ordinance no. 460/75, there had not yet been any regulation of DHTs by the National Congress through Law, which motivated the FCD to continue advocating for the effective regulation of the “professions”. The document also considered the existence of the training courses already functioning in the country, the entrance of a considerable number of these professionals in the market, the lack of discipline mechanisms for “professional” practice and the considerable concern then existing in the dental professional regarding this issue.

It is important to state that the FCD does not have the power to legislate on such matters, although it has sought to regulate the work of these professionals. Only the federal government can pass Laws that establish the qualifications and conditions for professional practice, as subsequently occurred in 2008, when Law 11.889 was enacted. The Law regulates the professional practice of OHTs in Brazil [8,49].

In the second half of the 1980s, some national associations positioned themselves against the regulation of the OHT profession and even requested the closure of the existing courses. The followers of these associations claimed that such technicians degraded dentistry, would aid public services in increasingly damaging the job market for dentists and would encourage the formation of false professionals. All of these were said to undermine the dignity of the dentistry profession [50]. The position generated a “Manifesto of Repudiation” divulged by the groups that defended the inclusion of OHTs on the dental team. This harks back to the theory put forth by Abbot [15] that there is a permanent dispute in dentistry in which the dentists aim to maintain their professional jurisdictions and monopolise their particular field of labour.

One of the triggering factors for the professional legitimization of OHTs in Brazil was the role of the public healthcare system [4], which was established in the 1988 constitution [9] during the process of democratization and was subsequently regulated through the Organic Health Law [51-53]. This healthcare system establishes the development of human resources for the professionalization of workers in the primary care network in order to reorient and qualify professional practice.

With the consolidation of the Brazilian public healthcare system, the Family Health Strategy (FHS) was adopted and put into operation through the implantation of multi-professional teams in primary health units. This model follows the fundamental attributes of primary health care, such as accessibility, breadth, integrality and coordination [5].

With regards to dentistry, there has been a perceptible intensification in the training and hiring of auxiliary personnel in the public sector, especially since the inclusion of Oral Health Teams in the FHS, thereby offering new prospects for the legal consolidation of the occupation [54]. The FHS, which involves a multi-professional team, is responsible for a defined geographic area, where it conducts promotion, protection, prevention, recovery and rehabilitation actions aimed at individuals and families in an integrated and continuous fashion.

The inclusion of OHTs facilitates the FHS in obtaining greater gains in health for the population, improving the oral health status of Brazilians, guiding oral healthcare practices as envisaged by the public healthcare system and ensuring the progressive access of families residing in the areas of FHS coverage to promotion, prevention and curative-restorative oral health actions [55].

The National Primary Healthcare Policy was approved in 2006 to regulate primary care as a healthcare model in Brazil. The policy united, in a single document, all the structural elements necessary for the organization of health services. This policy maintained the previous structure of the Oral Health Teams and the inclusion of DHTs. Importantly, this policy provides a financial incentive for municipalities to incorporate the OHTs in its Oral Health Team [56].

The Brazilian National Oral Health Policy, “Brazil Smiling”, which was launched in 2004, united a series of oral health actions directed at patients of all ages. This program encompasses both prevention-promotion actions and clinical care, considering oral health to be a healthcare model composed of different levels [6,57].

“Brazil Smiling” proposes joint work between the Oral Health Teams (including OHTs) and Family Health Teams. The program also aims to make viable public policies that ensure the implantation of fluoridated water in municipalities and other collective health actions [58].

In this context, the inclusion of the OHTs on the Oral Health Team within the SFH is a key element aiding healthcare and broadening access to healthcare services in a population with marked prevalence of dental caries and edentulism, especially among adults [57].

### Law 11.889: professionalization?

The importance of defining a day-to-day reference for workers in oral health should be stressed. This requires the building of an identity through regulating institutional action. New technologies, the increase in organizational complexity and growth in social demands underscore the need for professionalization through the regulation of specific professional fields [48].

The reasons that led to the delay in the approval of the OHT Law in Brazil and its main points in the current debate are discussed below.

In 1993, the Brazilian Congress approved a bill, which the president subsequently vetoed. Arguments against the bill charged that the regulation of such professionals would restrict the job market, delimit the field of action, discourage professional improvement and impede full hiring freedom. The veto was based on the fact that the project incurred excessive regulation of an activity that does not imply advanced knowledge. The application of the bill would determine the unnecessary creation of yet another professional board with new training and a restrictive market [59].

The veto was likely due to the decision of the FCD, which theoretically restricted the task attributions of the then-denominated DHTs in that same year, principally “to avoid the overlapping of activities between auxiliaries and dentists ”. This measure was reinforced by a corporative, mercantilist stance of dental councils at the time [60]. The necessity of overcoming the traditionalism made it difficult to build oral health teams in Brazil.

A number of bills were subsequently drafted for the regulation of the OHT profession, but a bill was not finally enacted until 2008 [8], more than 30 years after the establishment of the functions in Ordinance 460/75. The biggest problem was the struggle regarding skills. The issue always arises when a non-dentist has access to the oral cavities of patients. Neither the hygienists in the United States of America nor the dental nurses in New Zealand were easily accepted when these professions first emerged. In Brazil, the corporations have worked to restrict the activities of these workers since the 1940s. The federal regulation of the OHT resulted from wide-ranging negotiations involving different actors and stakeholders, representing diverse associations and movements of staff and technical areas, unions, federations and organizations of the dental profession; in addition to advice from professional bodies, health systems and educational institutions in the country [61]. Dentists who belong to an established profession, continue occupational strategies that centre on the protection and maintenance of boundaries [12,62,63]. In Brazil, with a high proportion of dentists, it is not difficult to understand the more frequent occurrence of jurisdictional disputes. Considering the dynamic movement toward professionalization [62], OHTs have moved their jurisdiction towards an area where they are “welcomed/needed/accepted”.

Based on the experience reported by Dunning [23] and confirmed by dozens of subsequent experiences throughout the following decades, the ideal technician is one with the necessary skills for the insertion of restorative materials in dental cavities previously prepared by the dentist, as well as the condensation, sculpturing, finishing and polishing of restorations [23].

The absence of any step of the restorative procedure would imply technical limitation, which diminishes the ability to compare Brazilian OHTs with internationally established experiments. According to Law 11.889 [8], an OHT can only perform the insertion and condensation of restorative materials in cavities prepared by a dentist. In other words, there is a division of labour and the interruption of its technical directionality, characterized by less contact with the oral cavity of the patient on the part of the OHT, which thereby defends entrenched interests. This is the sole activity delegated to OHTs, which is similar to the activities performed by the dental nurses of New Zealand. Qualification could result in wasted time, as it would not afford a dentist the freedom to perform another activity.

In the realm of individual preventive procedures, “performing biofilm removal based on the technical indication defined by the dentist” has led to discussion of Law 11.889, as the word “biofilm” does not specify the consistency of bacterial plaque, which may or may not be mineralized [64]. The result has been a huge debate regarding the legality of tartar removal by OHTs.

For community actions, the Law makes implicit a broad use of technicians, which specifies the work conditions of OHTs as multiplying agents or even supervisors with the stipulation “participate in educational actions for health promotion and the prevention of oral diseases”, as it does not impose specific prohibitions. Despite the lack of specific prohibitions, for the activities in which OHTs work directly with the oral cavity of the patient, the direct supervision of the dentist is indispensable.

This last issue takes us to the actual professionalization of OHTs. Autonomy is presented as an important attribute of a profession. This means that a professional exercises authority in his/her field of work without interference from others. Moreover, a professional is given the right to control the work itself and to regulate oneself [13]. OHTs in Brazil do not exercise autonomy and self-regulation, as they are dependent upon the direct or indirect supervision and action of the dentist. This situation, therefore, does not offer the means for a truly free professional. Power and autonomy only exist for a profession when legitimatized by society and regulated by Law [11]. In the case of the OHT, even the regulation has not fully enabled this.

In some states of the Unite States of America, such as Colorado and California, dental hygienists have the permission to execute clinical procedures without the supervision of the dentist. They can even set up their own offices and exclusively offer the dental hygiene services [31,34]. In The Netherlands, Norway, Switzerland (4 cantons), Sweden and Denmark, dental hygienists work in wholly independent practice. In the UK, dental hygienists can work in their own practices, but can only accept patients referred by a dentist [29]. Law 11.889 prohibits OHTs from exercising their activities in an autonomous fashion. In Brazil, considering the social conditions and the high number of dentists, this liberal practice could have more impact on the market than the improvement of the oral health of the population.

The passage of this Law may be seen as progress, as the occupation has now been nationally recognised, preventing the Federal Council of Dentistry from limiting professional OHT functions. Moreover, the difficulty of modify an existing Law can prevent the eventuality, with scientific advances, of new functions being performed by OHTs.

It is also important to stress that, although regulated, OHTs do not have the right to vote on their enrolled board and do not even have their own union. This again reflects the monopoly of dental interests in in Brazil. In the United States America, for instance, dental hygienists are organized in associations throughout all the states and have had a national association since 1923 that fights for their rights. They also have their own publications and participate in conferences and seminars, receiving complete support from dentists through the American Dental Association. The same occurs in England, Canada and Australia [1,2,22,65].

### Public health: an important field for the inclusion of OHTs

From the late 1970s to the early 1990s, faced with the consequences of the social security crisis in Brazil under the marked influences of the Alma Ata Declaration and the Health Promotion paradigm as well as under the influences of the sanitary reform and the creation of the Brazilian public healthcare system, the movement for the professional consolidation of OHTs signified an important change in the organization of oral health work. Despite the importance of this period, there are virtually no reports or reviews examining this issue.

However, during the same historical period, the new proposed profile and incorporation process of OHTs in public healthcare services may be translated as an effort to both build a new way of implementing dentistry-related practices in the field of public health as well as the inclusion of the profession in the model proposed by the public healthcare system [66]. This process signified the possibility of broadening clinical service offerings. In addition, it represented an important benchmark in the reorganisation of the work process in oral health within the teamwork-structuring model [67].

The process of OHT professionalization has been marked by internal ideological conflicts within the field of dentistry that question the competency of these professionals regarding the exercise of technical interventions in the oral cavity [68]. At the same time, the incorporation of OHTs in Oral Health Teams initially resulted in a productivist model, with a predominance of clinical actions as the main focus of the practice [66,69]. However, with the advance of the decentralization process of the Brazilian public healthcare system, the reorientation of the healthcare model based on primary care, the adoption of the Family Health Strategy and the issuance of Ordinance 648 in 2006—reinforcing the principles of primary care and redefining the functions of the diverse professionals of the health teams—a new horizon has opened in all sectors that constitute the Brazilian public healthcare system [56,70]. Since then, the importance of these professionals in the public health realm, performing general health actions, began to be considered a key point for the consolidation of OHTs among Brazilian professions.

Currently, the driving force for the training of Oral Health Technicians in Brazil is the National Oral Health Policy. OHTs involved in the Oral Health Teams seek to consolidate the practice directed toward the humanization of care. Thus, beyond clinical work, OHTs participate in the planning, follow up and assessment of actions developed within the area of coverage of the basic family health units, including identifying the needs and expectations of the population with regard to oral health; encouraging and executing health promotion measures, as well as educational and prevention activities; organising the work process in accordance with the guidelines of the Brazilian public healthcare system; sensitizing families with respect the importance of oral health for the maintenance of overall health; scheduling and performing in-home visits based on the identified needs; and developing inter-sector actions for the promotion of oral health [56].

Through their inclusion in the public healthcare system, OHTs have increasingly changed their work profile. As multiplying agents directed toward health promotion and working with a multi-professional team, the actions of OHTs have gained prominence. This tends to promote greater confidence in OHTs on the part of public and results in an increase in social prestige and credibility. According to Abbott [15], these factors are essential to the consolidation of a profession.

Table 1 displays the chronology of OHTs in Brazil.

**Table 1:** Chronology of OHTs in Brazil

Decade	Type of dental auxiliary	Actions	Reigning oral health idea	Profession elements
1950s and 1960s	DHA	Individual preventive-educational actions	Incremental System	Not regulated
1970s and 1990s	DHT	Individual and collective preventive-educational actions; restorative care	Simplified Dentistry	Regulated by educational law and FCD Supervision of the dentist is mandatory
2000s to present	OHT	Health promotion with tendency toward a reduction in restorative care	Brazil Smiling	Regulated by federal law The direct and indirect supervision of the dentist is mandatory

## Final considerations

Although OHTs in Brazil have a longstanding history, legal recognition of this occupation is recent.

Beginning in the 1950s, Brazil worked with dental auxiliaries with a profile similar to that of OHTs. The DHA proposed by SESP was a type of assistant for promoting individual prevention and performing rudimentary work with instruments on schoolchildren.

Beginning in the 1970s, the demand for health care, the simplification of techniques and new orientations that occurred in the education realm with the implantation of high school professionalization levels were the main reasons for the issuance of Ordinance 460/75, which created the DHT profession. DHTs were skilled with an array of functions, with a blend of characteristics of three basic types of professions: dental assistant, hygienist and dental nurse.

In 1988, with the institutionalization of the Brazilian public healthcare system, new forms of healthcare planning and management were required. In the 21^st^ century, with oral health included in the Family Health Strategy, OHTs have become fundamental actors in health promotion and disease prevention actions.

Despite this, Brazilian Law 11.889 was only enacted in 2008. Since the emergence of this type of dental assistant in Brazil, there have been technical and corporative limits on auxiliary practices encouraged by mid-level professionals and dentists. The associations and federations are divided on this impasse. There are some polemical points in this Law, such as the clinical functions of OHTs. Despite the criticisms of the Law, it is necessary to recognise its importance. Due to a federal Law, OHTs have come to legally “exist” and can more easily organise themselves as a labour force and fight for their rights.

One may wonder whether OHTs are truly professionalized, as they only exercise their activities under the supervision of the dentist, whether directly or indirectly. In many countries, this supervision is not necessary, and a dental technician can even open his/her own office. However, we insist that it is not easy to compare the theoretical framework of the sociology of professions [11,12,15] to intermediate-level occupations. Moreover, is not easy to predict the impact (if any) of the autonomy of OHTs over the oral health of Brazilian population.

The lack of sufficient credibility in the eyes of public is another challenge for this new oral health technician profession. The population is not as familiar with OHTs as desired and this is one of the elementary points for the development of a profession. It is quite likely that, with the increasingly accentuated inclusion of these professionals in the Brazilian public healthcare system, such recognition is closer to becoming reality.

It is important to research the professional aspirations among OHTs, or in other words, their professional plans [12,62]. Quantitative and qualitative methodology could be helpful in studying the strategies used by OHTs to achieve a certain social status as a “profession”, the historical gaps concerning their professionalization process and their actual role and activies in Brazilian Public Healthcare System.

Despite some dilemmas and the difficulties of classified OHTs as a profession, the actual regulation of the oral health technician profession in Brazil gives us a chance to enhance the development of our public healthcare system. In this way, health teams could achieve a professional position that both serve the public and are recognised by them for their contributions.

## Competing interests

The authors declare that they have no competing interests.

## Authors’ contributions

CASO carried out the review of literature, participated in the conception and design of this review. MAFW participated in the conception and design of this review. SDL and MHNGA conceived of the study, participated in its design and coordination. All authors helped to draft the manuscript, read and approved the final manuscript.

## Acknowledgements

This manuscript was sponsored by Fundação de Amparo à Pesquisa do Estado de Minas Gerais - FAPEMIG.
